# Establishing the Clinical Utility of Glucagon-Enhanced MRCP for Improved Hepatopancreatobiliary Assessment

**DOI:** 10.3390/jcm14175962

**Published:** 2025-08-23

**Authors:** Abdel-Rauf Zeina, Oren Shibolet, Mohamed Garra, Randa Taher, Oren Gal, Michael Oster, Rawi Hazzan, Ahmad Mahamid, Fadi Abu Baker

**Affiliations:** 1 Department of Radiology, Hillel Yaffe Medical Center, Hadera 38100, Israel; 2 Rappaport Faculty of Medicine, Technion Israel Institute of Technology, Haifa 3525433, Israel; 3 Department of Gastroenterology and Hepatology, Tel Aviv-Sourasky Medical Center, Tel Aviv 64239, Israel; 4 Gastroenterology and Hepatology Department, Hillel Yaffe Medical Center, Hadera 38100, Israel; 5 Liver Clinic, Clalit Health Services, Nof Haglil 17601, Israel; 6 Azrieli Faculty of Medicine, Bar-Ilan University, Safed 1311500, Israel; 7 Department of Surgery, Carmel Medical Center, Haifa 3436212, Israel

**Keywords:** glucagon, magnetic resonance cholangiopancreatography, biliary tree, pancreatobiliary imaging, diagnostic enhancement

## Abstract

**Background:** Magnetic Resonance Cholangiopancreatography (MRCP) has continuously evolved to enhance visualization capabilities. However, diagnosing biliary ductal system pathology, particularly early primary sclerosing cholangitis (PSC), remains challenging. This study investigates the influence of intramuscular glucagon (IMG) administration on final image quality and pancreatobiliary ductal system diameter in MRCP. **Methods:** Forty patients (57.5% female; average age 34.45 ± 8.2) referred for Magnetic Resonance Enterography (MRE) underwent MRCP before and 8–12 min after IMG administration. Two independent MRI specialists analyzed Coronal T2-weighted fast spin-echo high-resolution 3D MRCP images quantitatively and qualitatively. Quantitative assessments involved measuring the transverse diameter of five specific biliary duct structures (inferior, mid, and upper common bile duct; right and left hepatic ducts) and three pancreatic duct segments (head, body, and tail). The qualitative evaluation used a five-point Likert-type scale (1 = perfect visualization; 5 = not visible) for the predefined segments. Interobserver variation was assessed using the Intraclass Correlation Coefficient (ICC). **Results:** Following IMG administration, the diameters of all corresponding biliary and pancreatic segments significantly increased, with consistently strong interobserver agreement demonstrated pre- and post-IMG administration. Moreover, in qualitative analysis, post-IMG administration scores indicated a significant decrease (*p* < 0.01) in visualization scores, signifying improved visualization at all corresponding points for both radiologists compared to the pre-glucagon administration scores. The ICC scores pre- and post-IMG administration demonstrated moderate to strong agreement. **Conclusions:** IMG administration improves MRCP imaging parameters by increasing ductal diameters and enhancing biliary tree visualization, underscoring its potential to detect subtle or early pathological changes.

## 1. Introduction

Magnetic Resonance Cholangiopancreatography (MRCP) provides an accurate non-invasive, high-resolution imaging modality [1,2,3]. The widespread acceptance and application of MRCP in diverse hepatobiliary conditions underscore its versatility and reliability. It is widely used for the diagnosis of choledocholithiasis biliary cysts, congenital anomalies, structural variations and cancers of the biliary tract, contributing substantively to a comprehensive non-invasive assessment of the biliary tree [4,5,6,7]. Its significance in diagnosing primary sclerosing cholangitis (PSC), surpassing Endoscopic Retrograde Cholangiopancreatography (ERCP) in efficacy and reducing associated complications, is well-documented [8,9]. Importantly, unlike MRCP, ERCP is an invasive procedure that requires endoscopy and often conscious sedation. It also carries risks such as post-procedure pancreatitis due to cannulation of the sphincter of Oddi and contrast medium injection. Furthermore, ERCP visualizes the biliary system in a retrograde fashion and, requiring manual or hydraulic pressure, can sometimes fail to fully delineate the entire intrahepatic biliary system. Additionally, MRCP’s capacity to unveil structural abnormalities and delineate changes in pancreatic duct caliber is favored over CT for evaluating and following up on pancreatic cystic neoplasms and other pancreatic pathologies [10,11].

Despite these strides, challenges persist in detecting microcholedocholithiasis, particularly in non-dilated CBD, and in the preoperative evaluation of jaundiced patients referred for cholecystectomy. For PSC cases, challenges are heightened in patients with cirrhosis and early subclinical disease due to subtle changes in the biliary tract and suboptimal visualization of peripheral intrahepatic branches [12].

Ongoing research seeks to enhance MRCP’s sensitivity, addressing limitations and broadening its diagnostic scope. Technological advancements, including higher field strength and accelerated scanning protocols, aim to refine MRCP’s diagnostic precision. Central to this refinement are strategies to augment its visualization potential. Pharmacological agents, including opiates, secretin, and their combinations, have been explored to enhance imaging precision. Opiates, such as morphine, induce sphincter of Oddi contraction, enhancing ductal distention [13,14]. Secretin, a gastrointestinal hormone, stimulates the exocrine pancreas leading to bicarbonate-rich fluid secretion and subsequent dilatation of the pancreatic ductal system, contributing to improved MRCP image quality [15]. The concurrent administration of opiates and secretin, investigated as a strategy to synergistically augment biliary tree visualization, may, however, be complicated by administration timing, adverse events, and procedure elongation.

In the present investigation, our objective was to conduct a comprehensive qualitative and quantitative assessment of the potential augmentation effect of glucagon, known for its ability to increase bile flow, on the visualization of the pancreatobiliary tree in suspected Inflammatory Bowel Disease (IBD) patients referred for Magnetic Resonance Enterography (MRE). This hypothesis utilized the fact that MRE protocols typically incorporate glucagon administration.

## 2. Methods

### 2.1. Patient Selection

This case-controlled study design, where each patient acted as their own control, compared MRCP images obtained before and after intramuscular glucagon (IMG) administration. Forty consecutive, non-selected subjects (age ≥ 18 years) with known or suspected Inflammatory Bowel Disease (IBD) referred for Magnetic Resonance Enterography (MRE) underwent MRCP before and after intramuscular glucagon (IMG) administration, in the Imaging Institute of Hillel Yaffe Medical Center throughout the years 2022–2023. All patients were informed about the study protocol and provided signed informed consent. Exclusion criteria included age under 18, a history of biliary tract intervention or surgery, uncontrolled diabetes, severe chronic kidney disease (GFR < 50), and the use of opiates or opiate derivatives. Demographic and clinical data, including the patients’ age, gender, history of IBD (such as Crohn’s disease and ulcerative colitis) and the diagnosis of PSC, were collected from the medical files. This prospective study was approved by our institutional review board (IRB num 22-992 HYMC).

### 2.2. Imaging Technique

All subjects underwent combined MRE and MRCP. All MRE and MRCP studies were performed on a Philips Achieva 1.5T, Philips Medical Systems Nederland B.V. in Best, The Netherlands, using a 16-channel phased array body surface coil. On the day of the examination, 50–60 min before, each subject received an oral solution of biphasic contrast medium, previously prepared by dissolving 250–300 mL of 18% mannitol in 1500 mL of water in order to achieve a 3.5–4% solution. The standard MRE protocol consisted of the following imaging sequences and parameters: Coronal 2D balanced turbo-field echo (BTFE), coronal and axial T2W single-shot turbo spin echo (SSh-TSE), axial T2W single-shot turbo spin echo SPAIR (SSh-TSE-SPAIR), coronal and axial T1W high-resolution isotropic volume (THRIVE) before and after intravenous administration of 0.15 mL/Kg of gadolinium diethylenetriamine penta-acetic acid (Gd-DTPA) (Gadolinium Diethylenetriamine Penta-Acetic Acid (Gd-DTPA), Catalog number: 381667, Sigma-Aldrich, Burlington, MA, USA), diffusion weighted images in the coronal planes (b = 0/100/800). MRCP images were acquired in coronal and coronal oblique planes with a heavily T2-weighted, fast spin-echo pulse sequence, using respiratory triggering, before and 9–14 min after IM administration of glucagon (1 mg) (GlucaGen^®^ HypoKit, NDC: 0169-7065-15, Novo Nordisk A/S in Bagsvaerd, Denmark), with the following imaging parameters: effective echo time 1400 ms; repetition time 4800 ms; acquisition matrix 256 × 256; field of view 28 × 28 cm; flip angle 90°; slice thickness 1.2 mm; gap 1.1 mm. Parallel imaging with an acceleration factor of 2 was also utilized. The mean acquisition time of the MRCP sequence, including respiratory compensation, was 7 ± 0.5 min. All subjects were observed for drug-related side effects for at least 30 min after the study.

### 2.3. Imaging Analysis

**Figure 1:**
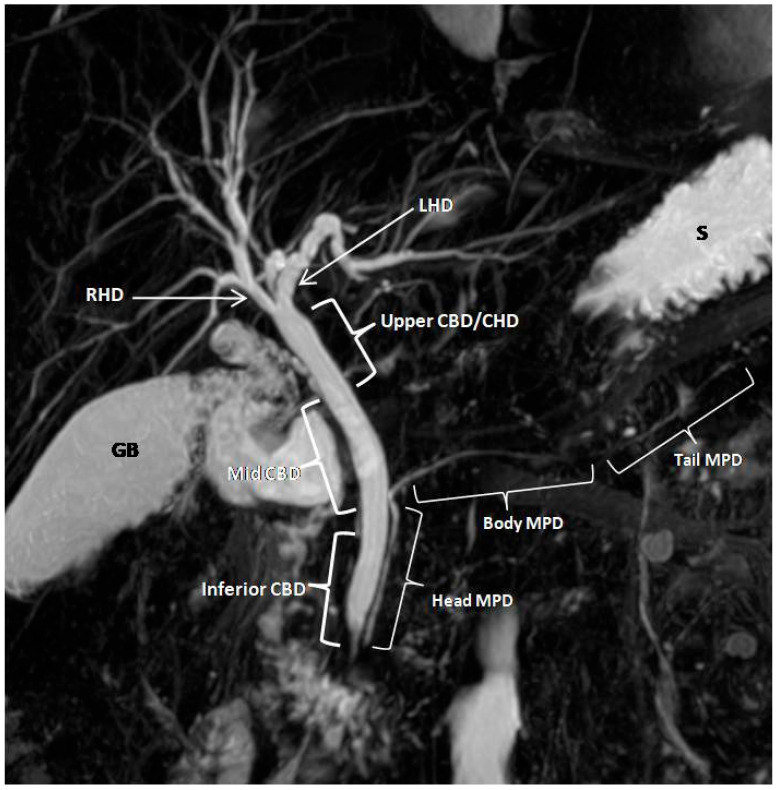
Coronal maximum intensity projection (MIP) image generated from respiratory-triggered 3D high-resolution MRCP sequence showing the pancreaticobiliary tree ductal segments included in the quantitative and qualitative analysis. CBD, common bile duct; CHD, common hepatic duct; LHD, left hepatic duct; RHD, right hepatic duct; MPD, main pancreatic duct; GB, gallbladder; S, stomach.

Coronal T2-weighted fast spin-echo high resolution 3D MRCP images and coronal maximum intensity projections generated from the same data were used for the study analysis in all subjects. Two experienced abdominal radiologists, blinded to clinical data, conducted the image analysis. For quantitative analysis, transverse diameters of the common bile duct at three levels (Inferior CBD, Mid CBD and Upper CBD), right and left hepatic ducts (1 cm proximal to the confluence of the right and left hepatic ducts), and the main pancreatic duct at three levels (Head MPD, Body MPD and Tail MPD) were measured at corresponding points on the images before and after glucagon administration (Figure 1). Qualitative assessment was based on visual evaluations. The depiction of predefined segments of the biliary and pancreatic ductal tree was scored independently by the two radiologists on a Likert-type 5-point scale (Table 1) adopted from Asbach et al. [16]: 1 = perfect visualization of entire ductal structure; 2 = most of the ductal structure visualized; 3 = ductal structure partially visible; 4 = detection of ductal structure almost impossible; 5 = ductal structure not visible. Interobserver variation was assessed using Pearson’s correlation and the Intraclass Correlation Coefficient (ICC).

### 2.4. Statistical Analysis

Quantitative assessment of the pancreatobiliary duct measurements pre- and post-intramuscular glucagon (IMG) administration was performed utilizing the paired t-test to analyze changes in ductal diameters. For qualitative evaluation of visualization, the non-parametric Wilcoxon signed-rank test was employed to compare Likert-type scale scores before and after IMG administration to ensure the validity of the findings, particularly in the presence of any outliers or if the qualitative data was not normally distributed. The assumptions for statistical tests were verified. Interobserver agreement was determined using the Intraclass Correlation Coefficient (ICC). All statistical analyses were conducted using IBM SPSS Statistics software version 28, with a predetermined significance threshold set at *p* < 0.05.

## 3. Results

### 3.1. Patients’ Characteristics

The MRCP was successfully performed in all of the 40 subjects who were enrolled in the study. Of them, 23 were female (57.5%), with an average age of 34.45 ± 8.2. Notably, none of the participants had a history of diabetes, were under diabetic medication, or presented with chronic kidney disease [mean Glomerular Filtration Rate (GFR) was 98 (range: 84 to 132)]. The mean interval between glucagon administration and the subsequent MRCP scan was 11.2 min (range: 9.2–13.4 min). The mean acquisition time of this sequence, including respiratory compensation, was 7 ± 0.9 min. The overall mean duration of the examination, encompassing both MRCP and MRE, was 40 ± 7.2 min.

Concerning MRE diagnoses, 19 patients (47.5%) exhibited evidence of changes in the small intestine or colon indicative of active inflammation. In terms of MRCP diagnoses, two patients (5%) were identified with PSC, and an additional two patients (5%) were diagnosed with gallbladder stones. In the remaining subjects, no abnormalities were detected.

### 3.2. Quantitative Analysis

In the quantitative analysis, the mean maximum diameter of the biliary segments, as measured by both radiologists, consistently increased following glucagon administration. All corresponding points demonstrated a significant diameter increase (*p* < 0.001), with the most substantial increases observed in the right and left hepatic ducts. The high ICC values and R ratios remained consistent, indicating strong agreement between the two radiologists. These findings are presented in detail in Table 2.

In the pancreas, the mean maximum diameter of the main pancreatic duct’s head, body, and tail segments, as measured by both radiologists, showed a significant increase following glucagon administration. For both radiologists, a significant diameter increase was demonstrated at all corresponding points post-glucagon administration (*p* < 0.001). The ICC values and R ratios were consistently very high, highlighting a strong interobserver agreement before and after glucagon administration. These findings are presented in detail in Table 3.

Our quantitative analysis demonstrates a consistent and significant effect of glucagon administration across the entire pancreatobiliary system. The significant increase in ductal diameters observed in the biliary tree was mirrored by a significant increase in all segments of the pancreatic duct. These findings underscore glucagon’s consistent physiological effect on both ductal systems. The consistently high interobserver agreement for all quantitative measurements further reinforces the reliability and reproducibility of these findings.

### 3.3. Qualitative Analysis

In the qualitative analysis, both radiologists consistently reported a significant decrease (*p* < 0.001) in visualization scores following IMG administration, signifying a notable improvement in visualization at all corresponding points. This enhancement was particularly pronounced in the peripheral intrahepatic biliary segments, where scores showed the most significant decreases. The ICCs and R ratios pre- and post-glucagon administration demonstrated moderate to strong agreement across all segments, confirming the reliability of these qualitative findings. These findings are presented in detail in Table 4.

In the qualitative assessment of pancreatic segments, both radiologists reported a notable improvement, with a significant decrease (*p* < 0.001) in visualization scores at all corresponding points following glucagon administration. The ICCs and R ratios pre- and post-glucagon administration demonstrated moderate to strong agreement across all segments. These findings are presented in detail in Table 5.

Overall, our qualitative analysis consistently demonstrated that glucagon administration significantly improved visualization throughout the entire pancreatobiliary system. The enhancement was observed from the main ducts down to the smaller peripheral intrahepatic segments and across all pancreatic segments, providing strong qualitative support for the use of glucagon in MRCP protocols.

## 4. Discussion

**Figure 2:**
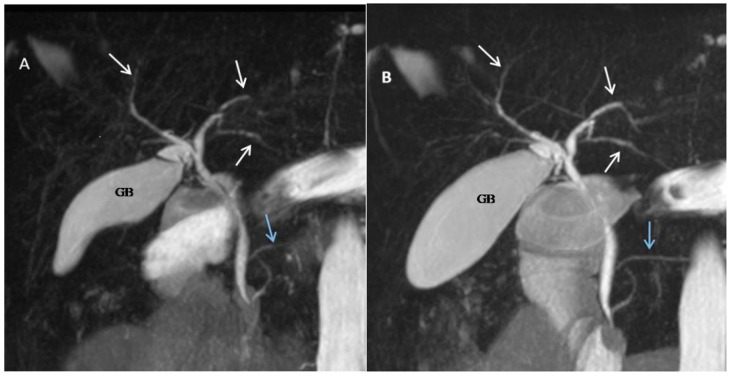
A 29-year-old female with Crohn’s disease. Coronal maximum intensity projection images obtained before (**A**) and after (**B**) IM administration of glucagon demonstrate improved visualization of the intrahepatic biliary ducts (white arrows) and the main pancreatic duct (blue arrow) post-glucagon administration. Note the increased diameter of the pancreatobiliary tree and gallbladder (GB).

The present study provides valuable insights into the augmentation of MRCP outcomes through the inclusion of glucagon in the imaging protocol. The enhancement in both quantitative and qualitative visualization across various anatomical points, as evaluated by two abdominal radiologists with a high level of concordance, signifies the potential of IMG to significantly enhance MRCP results (Figure 2). This improvement, reflected in reduced Likert-type scores and increased transverse diameters at predefined points, not only attains statistical significance but also establishes the clinical relevance of IMG in optimizing MRCP.

To comprehensively examine the mechanisms underlying the observed enhancements, it is crucial to explore the physiological effects of glucagon. This peptide hormone, secreted from the alpha cells of the pancreatic islets of Langerhans, is primarily recognized for its role in stimulating glucose production in the liver, thereby maintaining adequate plasma glucose concentrations. Beyond glucose regulation, glucagon also plays a significant role in hepatic lipid and amino acid metabolism [17]. Notably, studies have demonstrated that glucagon possesses the ability to increase bile flow and dilate the biliary tree [18]. Furthermore, its direct action on smooth muscle potentially reduces gastrointestinal peristalsis, possibly contributing to the mitigation of artifacts related to peristalsis on MRCP images [19]. Crucially, the well-tolerated nature of glucagon administration, coupled with the absence of reported adverse events, contributes to the safety profile of the protocol [20]. The minimal impact on the overall procedure duration further underscores the feasibility and practicality of integrating glucagon into routine clinical settings. These findings collectively position the proposed approach, involving glucagon administration preceding MRCP study, as a patient-friendly and efficient enhancement strategy, applicable in everyday diagnostic workflows.

Old anecdotal reports suggested that low-dose glucagon enhances cholangiography quality without side effects [21]. A 1983 study involving fourteen patients undergoing cholecystectomy found that intravenous glucagon consistently improved biliary tree visualization compared to a control group receiving saline, with no significant adverse effects observed [22].

In contrast, the study conducted by Van Hoe and colleagues (1998) failed to yield substantial enhancements in the visualization of the distal CBD subsequent to glucagon administration. Notably, this investigation employed intravenous glucagon injections, with a cohort of 30 patients allocated to each treatment group. Central to the inquiry was the examination of the Vater sphincter complex’s visual representation. However, the study faced limitations due to its use of older imaging techniques and uncertainties surrounding instances where visualization was not achieved, possibly linked to variations in breathing patterns during successive breath holds [23].

A study by Dalal et al. in 2004 [24] supported our findings, demonstrating the potential of glucagon to enhance MRCP visualization. This investigation, which involved 42 non-diabetic subjects with symptomatic choledocholithiasis, and glucagon was administered intravenously. The study, utilizing the half-Fourier single-shot turbo spin-echo (HASTE) sequence, showed improved visualization of the common bile duct and ampulla of Vater post-glucagon, with no reported adverse effects [24]. Additionally, our findings are supported by the 2007 study by Hellund et al. [25], which found that glucagon administration reduced artifacts from bowel movements and improved the visibility of the pancreatic duct [25]. In a 2009 study focusing on the combination of glucagon with opiates, 16 liver donor candidates were prospectively evaluated. That study demonstrated that the combined administration of intravenous low-dose morphine with intramuscular glucagon significantly increased the diameters of the biliary and pancreatic ducts and improved the visualization of non-dilated biliary ductal anatomy [26].

Advantages of our study include its rigorous methodology, comprehensive exploration of multiple predefined points on the pancreatobiliary tree, and a meticulous focus on both qualitative and quantitative measurements. The utilization of a case-controlled approach with patients acting as their own controls allowed for the standardization of techniques, minimizing bias from inter-patient anatomical variation. Furthermore, our study engaged two expert radiologists, demonstrating a high concordance in interobserver agreement. These strengths contribute to a more detailed and insightful understanding of the benefits of glucagon in MRCP protocols, surpassing the limitations of previous studies and enhancing the credibility of our findings.

**Figure 3:**
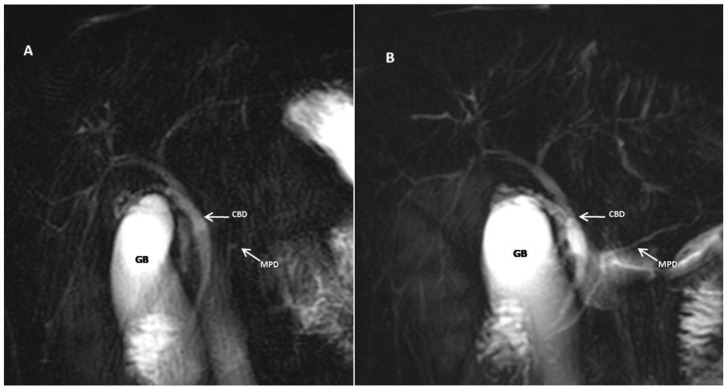
A 28-year-old female with ulcerative colitis and primary sclerosing cholangitis (PSC). Coronal MRCP images obtained before (**A**) and after (**B**) IM administration of glucagon. The multifocal strictures and dilatations of the intrahepatic bile ducts are more clearly depicted on the MRCP image obtained after the administration of glucagon (**B**). CBD, common bile duct; MPD, main pancreatic duct; GB, gallbladder.

The study’s strategic inclusion of patients with suspected IBD, specifically referred for MRE, aligns with the rationale behind the investigation. Leveraging the routine use of glucagon in MRE protocols, the study explored the augmentative effects of glucagon in MRCP. Given the heightened risk of PSC in patients with IBD, the combined MRCP–MRE approach may be considered as a comprehensive diagnostic strategy. This aligns with the imperative for early PSC detection in this population, where subtle biliary tract changes may indicate the onset of the disease. Glucagon’s role in enhancing visualization positions it as a valuable tool in MRCP protocols designed for the early diagnosis of PSC, particularly in cases with subtle manifestations. The detailed examination of two cases of PSC in the current cohort provides additional support for the study’s findings. The significantly more evident visualization and characterization of changes in the biliary tract in these cases underscore the specific applicability of glucagon in elucidating intricate alterations associated with PSC (Figure 3). This insight reaffirms the enhanced diagnostic capabilities that would be particularly relevant in scenarios where the subtleties of PSC manifestations demand heightened precision and clarity for accurate detection.

**Figure 4:**
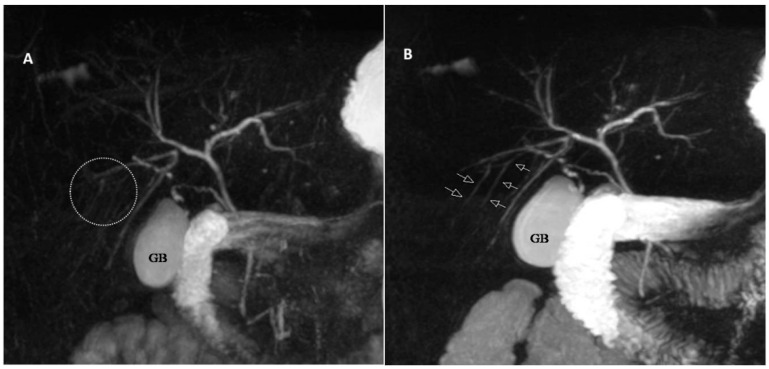
A 22-year-old female with abdominal pain and diarrhea. (**A**,**B**), Coronal maximum intensity projection images obtained before (**A**) and after (**B**) IM administration of glucagon reveal small intrahepatic ducts that were not visible before glucagon administration. The gallbladder (GB) volume is also increased and better visualized.

Moving beyond PSC, the study’s findings may extend to other disorders involving the gallbladder, biliary tree, and pancreatic tree (Figure 4). For example, the improved visualization could be particularly valuable in assessing subtle ductal changes associated with chronic pancreatitis or identifying small intraductal tumors like intraductal papillary mucinous neoplasms (IPMNs) of the biliary and pancreatic ducts. Glucagon’s role in improving visualization along the entire pancreatobiliary tree broadens its utility in MRCP, presenting it as a valuable adjunct for the comprehensive assessment of various hepatobiliary and pancreatic conditions. This positions glucagon as a potential enhancer in MRCP protocols. The potential clinical implications of this study are significant, as the observed improvement in visualization following glucagon administration may reduce the need for repeat investigations or the need for ERCP.

The visual evidence provided in our figures is central to our findings. Figure 2, for example, illustrates the significant qualitative and quantitative improvements in visualization post-glucagon administration, which aligns with our core findings on increased ductal diameters and enhanced visualization. Furthermore, Figure 3 provides a compelling example of the clinical utility of our protocol, showing how glucagon can more clearly delineate subtle pathological changes, such as the multifocal strictures and dilatations in a patient with PSC. Finally, Figure 4 highlights a key finding of our study: glucagon’s ability to reveal small intrahepatic ducts that were not visible in the pre-glucagon scan. This directly supports our conclusion about the potential of glucagon to detect subtle pathological changes.

The current study has several limitations that merit consideration. The study’s modest sample size, encompassing 40 patients, and the single-center design warrant cautious interpretation, as these factors may limit the generalizability of findings. Future research endeavors should consider larger cohort studies to validate and extend the implications observed in this investigation. Moreover, the limited cases with pathological findings may hinder the extrapolation of results to scenarios involving specific abnormalities. The absence of comparative groups and the selective focus on patients with suspected IBD undergoing MRE may limit broader applicability. Additionally, while the study incorporates a qualitative assessment through Likert-type scores, the absence of longitudinal follow-up limits insights into the durability and dynamic changes of the observed enhancements and warrants further investigations to refine understanding.

In conclusion, this study illuminates the potential of incorporating glucagon in MRCP protocols to enhance the diagnostic precision of disorders affecting the pancreatobiliary tree. The integration of MRCP with MRE, utilizing routinely administered glucagon, emerges as a valuable strategy for augmenting its diagnostic capabilities with an acceptable safety profile and without lengthening procedure time. These findings pave the way for advancements in MRCP protocols, promising refined diagnostic accuracy and improved visualization in hepatobiliary and pancreatic disorders.

## Figures and Tables

**Table 1:** Five-point grading scale for qualitative assessment of visualization of the ductal segments.

Score	Description
1	Perfect visualization of entire ductal structure
2	Most of the ductal structure visualized
3	Ductal structure partially visible
4	Detection of ductal structure almost impossible
5	Ductal structure not visible

Adopted from Ref. [16].

**Table 2:** Diameter measurements of biliary tract segments before and after intramuscular glucagon administration.

Measurements	Inferior CBD	Mid CBD	Upper CBD/CHD	RHD	LHD
Pre-Glucagon Administration					
Radiologist 1	1.17 ± 3.115	3.643 ± 1.46	3.792 ± 2.25	2.262 ± 1.25	2.70 ± 1.31
Radiologist 2	3.043 ± 1.14	3.548 ± 1.45	3.680 ± 2.22	2.193 ± 1.24	2.645 ± 1.30
R ratio	0.892	0.915	0.909	0.907	0.907
ICC	0.884 [0.879–0.894]	0.915 [0.911–0.917]	0.919 [0.918–0.919]	0.907 [0.904–0.908]	0.907 [0.904–0.909]
Post-Glucagon Administration					
Radiologist 1	3.640 ± 1.07	4.270 ± 1.45	4.385 ± 1.96	2.980 ± 0.94	3.435 ± 0.84
Radiologist 2	3.602 ± 1.03	4.143 ± 1.37	4.180 ± 1.84	2.927 ± 0.89	3.350 ± 0.79
R ratio	0.989	0.995	0.999	0.997	0.997
ICC	0.989 [0.979–0.994]	0.995 [0.991–0.997]	0.999 [0.998–0.999]	0.997 [0.994–0.998]	0.997 [0.994–0.999]
Statistical Significance Post- vs. Pre-Glucagon (*p* value)					
Radiologist 1	*p* = 0.004	*p* < 0.001	*p* < 0.001	*p* < 0.001	*p* = 0.002
Radiologist 2	*p* = 0.009	*p* < 0.001	*p* = 0.002	*p* < 0.001	*p* = 0.004

CBD—Common Bile Duct, CHD—Common Hepatic Duct, RHD—Right Hepatic Duct, LHD—Left Hepatic Duct, ICC—Intraclass Correlation Coefficient.

**Table 3:** Diameter measurements of pancreatic segments before and after intramuscular glucagon administration.

Measurements	Head	Body	Tail
Pre-Glucagon Administration			
Radiologist 1	0.88 ± 1.698	0.99 ± 1.495	0.95 ± 0.803
Radiologist 2	0.87 ± 1.652	0.98 ± 1.435	0.93 ± 0.808
R ratio	0.995	0.990	0.984
ICC	[0.991–0.997] 0.99	[0.981–0.995] 0.990	[0.970–0.991] 0.984
Post-Glucagon Administration			
Radiologist 1	0.98 ± 2.423	0.72 ± 2.120	0.72 ± 1.490
Radiologist 2	0.84 ± 2.315	0.66 ± 2.007	0.71 ± 1.395
R ratio	0.969	0.917	0.966
ICC	[0.921–0.977] 0.957	[0.843–0.953] 0.914	[0.936–0.982] 0.966
Statistical Significance Post- vs. Pre-Glucagon (*p* value)			
Radiologist 1 Pre vs. post	*p* < 0.001	*p* < 0.001	*p* < 0.001
Radiologist 2 Pre vs. post	*p* < 0.001	*p* < 0.001	*p* < 0.001

Head—Head of Main Pancreatic Duct, Body—Body of Main Pancreatic Duct, Tail—Tail of Main Pancreatic Duct, ICC—Intraclass Correlation Coefficient.

**Table 4:** Qualitative assessment scores of biliary tract segments visualization before and after intramuscular glucagon administration.

Visualization Segments	Inferior CBD	Mid CBD	Upper CBD/CHD	RHD	LHD	Intrahepatic
Pre-Glucagon Administration						
Radiologist 1	2.27 ± 0.68	2.40 ± 0.63	2.25 ± 0.67	2.65 ± 0.80	2.55 ± 0.68	2.95 ± 0.68
Radiologist 2	2.35 ± 0.70	2.33 ± 0.66	2.23 ± 0.77	2.65 ± 0.89	2.53 ± 0.82	2.83 ± 0.82
R ratio	0.655 [0.435–0.802]	0.791 [0.638–0.884]	0.877 [0.780–0.933]	0.929 [0.870–0.962]	0.814 [0.719–0.913]	0.668 [0.535–0.802]
ICC	0.656	0.791	0.885	0.934	0.856	0.782
Post-Glucagon Administration						
Radiologist 1	1.35 ± 0.53	1.45 ± 0.55	1.48 ± 0.55	1.63 ± 0.77	1.45 ± 0.55	1.83 ± 0.77
Radiologist 2	1.38 ± 0.59	1.28 ± 0.51	1.35 ± 0.53	1.55 ± 0.71	1.43 ± 0.55	1.91 ± 0.71
R ratio	0.718	0.555	0.638	0.707	0.536	0.653
ICC	0.715 [0.522–0.838]	0.553 [0.295–0.736]	0.637 [0.409–0.790]	0.705 [0.507–0.832]	0.536 [0.274–0.725]	0.651 [0.396–0.843]
Statistical Significance Post- vs. Pre-Glucagon (*p* value)						
Radiologist 1	*p* < 0.001	*p* < 0.001	*p* < 0.001	*p* < 0.001	*p* < 0.001	*p* < 0.001
Radiologist 2	*p* < 0.001	*p* < 0.001	*p* < 0.001	*p* < 0.001	*p* < 0.001	*p* < 0.001

CBD—Common Bile Duct, CHD—Common Hepatic Duct, RHD—Right Hepatic Duct, LHD—Left Hepatic Duct, ICC—Intraclass Correlation Coefficient.

**Table 5:** Qualitative assessment scores of pancreatic segments visualization before and after intramuscular glucagon administration.

Visualization	Head	Body	Tail
Radiologist 1	2.63 ± 0.77	3.05 ± 0.75	3.47 ± 0.75
Radiologist 2	2.68 ± 0.76	3.15 ± 0.80	3.60 ± 0.74
R ratio	0.872	0.926	0.854
ICC	0.872 [0.771–0.930]	0.923 [0.860–0.959]	0.854 [0.740–0.920]
Post-Glucagon Administration			
Radiologist 1	1.53 ± 0.72	1.88 ± 0.76	2.48 ± 0.75
Radiologist 2	1.60 ± 0.67	1.95 ± 0.71	2.48 ± 0.72
R ratio	0.875	0.841	0.906
ICC	0.873 [0.773–0.931]	0.840 [0.717–0.912]	0.905 [0.827–0.948]
Statistical Significance Post- vs. Pre-Glucagon (*p* value)			
Radiologist 1 Pre vs. post	*p* < 0.001	*p* < 0.001	*p* < 0.001
Radiologist 2 Pre vs. post	*p* < 0.001	*p* < 0.001	*p* < 0.001

Head—Head of Main Pancreatic Duct, Body—Body of Main Pancreatic Duct, Tail—Tail of Main Pancreatic Duct, ICC—Intraclass Correlation Coefficient.

## Data Availability

The data presented in this study are not publicly available due to patient privacy and ethical restrictions. However, data may be made available on reasonable request from the corresponding author.

## Abbreviations

2D—Two-Dimensional, 3D—Three-Dimensional, BMI—Body Mass Index, BTFE—Balanced Turbo-Field Echo, CBD—Common Bile Duct, CHD—Common Hepatic Duct, CT—Computed Tomography, ERCP—Endoscopic Retrograde Cholangiopancreatography, FOV—Field of View, Gd-DTPA—Gadolinium Diethylenetriamine Penta-Acetic Acid, GFR—Glomerular Filtration Rate, HASTE—Half-Fourier Acquisition Single-shot Turbo Spin-Echo, ICC—Intraclass Correlation Coefficient, IMG—Intramuscular Glucagon, IBD—Inflammatory Bowel Disease, LHD—Left Hepatic Duct, MRE—Magnetic Resonance Enterography, MRI—Magnetic Resonance Imaging, MRCP—Magnetic Resonance Cholangiopancreatography, MPD—Main Pancreatic Duct, PSC—Primary Sclerosing Cholangitis, RHD—Right Hepatic Duct, SD—Standard Deviation, SPAIR—Spectral Attenuated Inversion Recovery, SSh-TSE—Single-shot Turbo Spin Echo, SPSS—Statistical Package for the Social Sciences, TSE—Turbo Spin Echo.
